# Supplementary material for: Stretchy Electrochemical Harvesters for Binarized Self-Powered Strain Gauge-Based Static Motion Sensors
Source: Sensors (Basel). 2022 Jun 16;22(12):4542. doi: 10.3390/s22124542 (PMC9231270; doi:10.3390/s22124542)
Supplement: Supplementary file 1 [file sensors-22-04542-s001.zip › sensors-1767158-supplementary.pdf]

Supporting Information

Stretchy Electrochemical Harvesters for Binarized Self-Powered Strain Gauge-Based Static Motion Sensors

Hyeon Jun Sim, Jeeeun Kim, Jin Hyeong Choi, Myoungeun Oh and Changsoon Choi *

Department of Energy and Materials Engineering, Dongguk University, Seoul 04620, Korea

* Correspondence: cschoi84@dongguk.edu

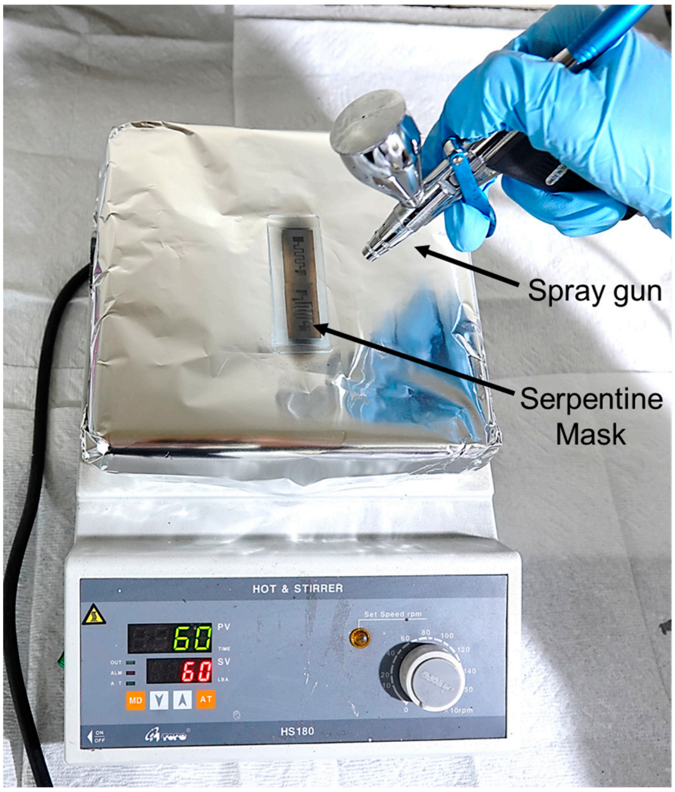

Figure S1. The optical image of spray-coating process for bilayer fabrication.

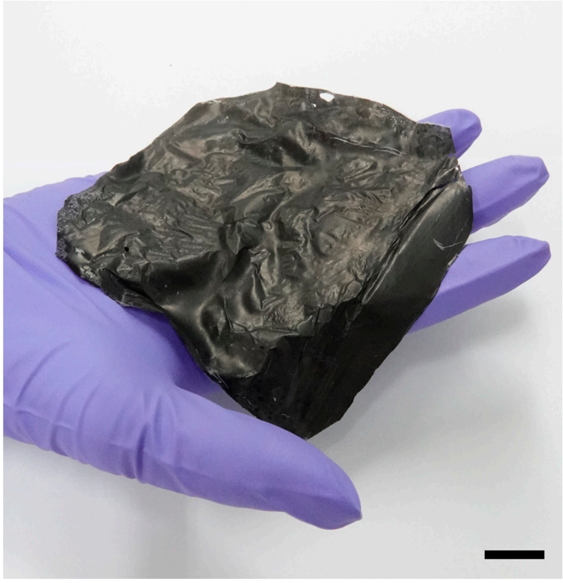

Figure S2. The optical image of MWNT/SEBS bilayer fabricated by spray-coating (scale bar: 1 cm).

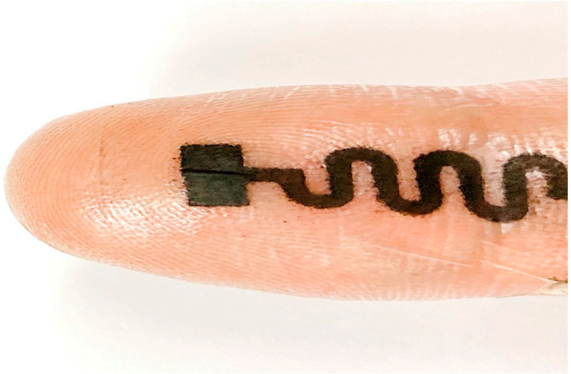

Figure S3. The optical image of serpentine patterned bilayer on the index finger.

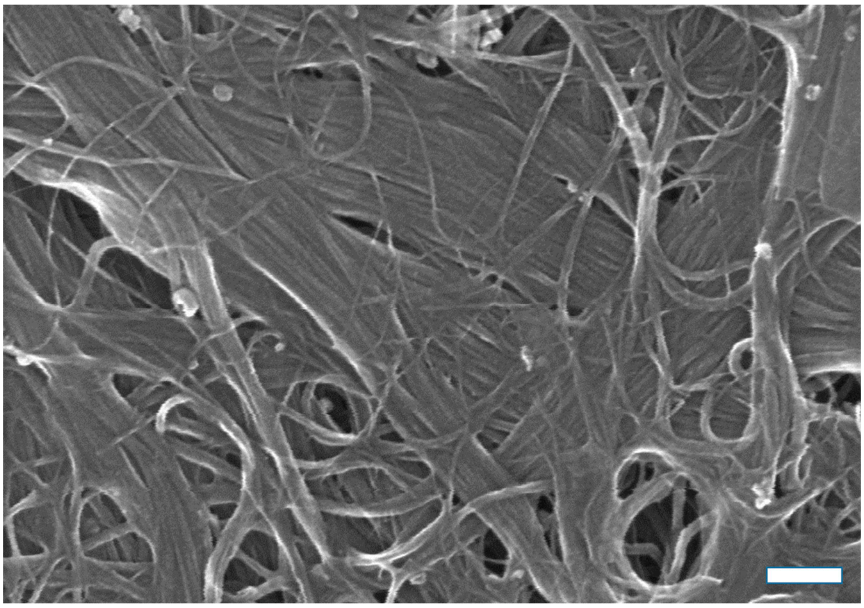

Figure S4. The SEM image of entangled multi-walled carbon nanotube (scale bar: 100 nm).

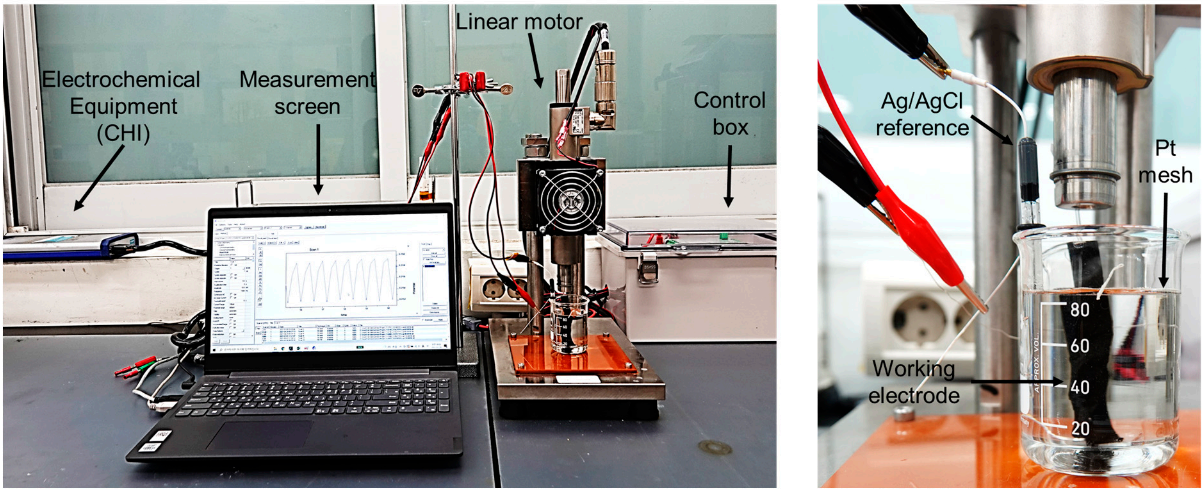

Figure S5. The experimental setting for measuring electrical energy from stretch-eletrochemical harvester.

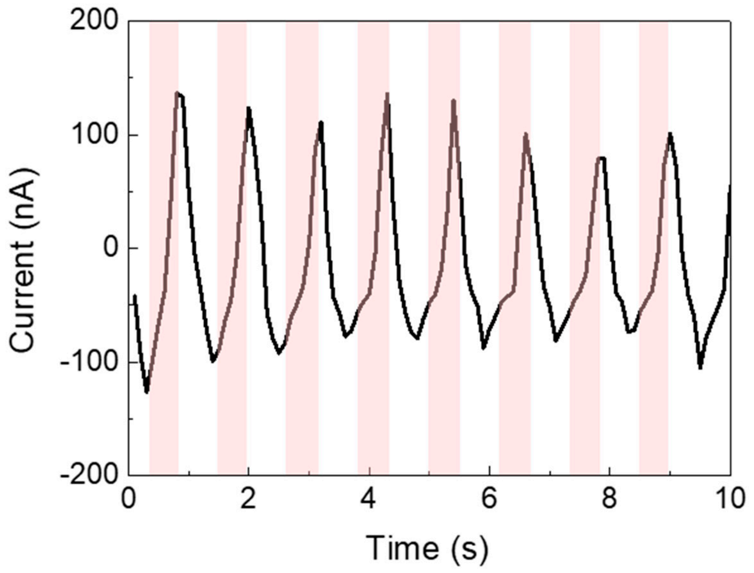

Figure S6. Short-circuit current with sinusoidal applied tensile strain. The red area is in a stretched state.

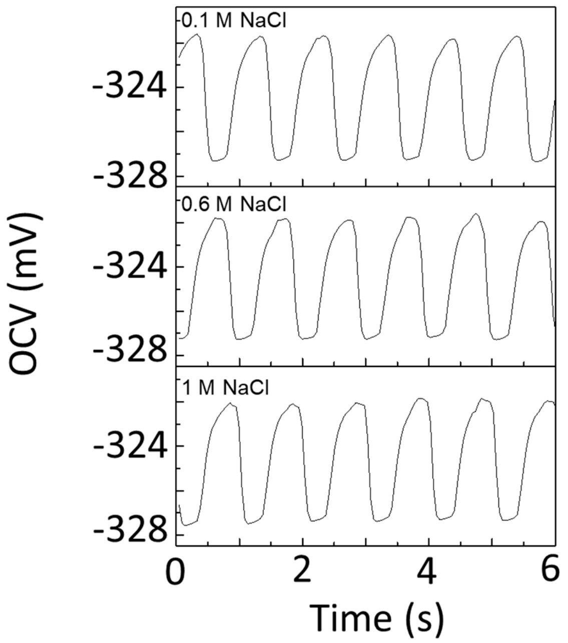

Figure S7. The open-circuit voltage with various NaCl concentration from 0.1M to 1M during 1-Hz sinusoidal 50% stretch.

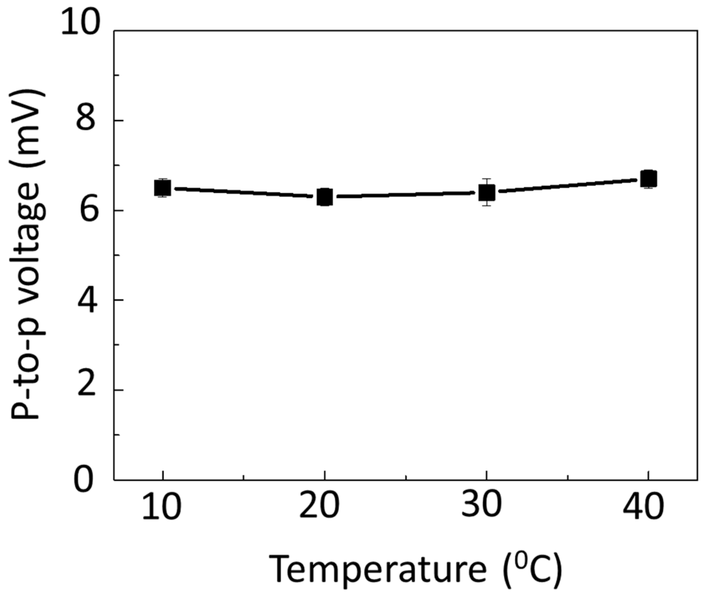

Figure S8. The open-circuit peak-to-peak voltage with various environmental temperature from 10 ^{0}C to 40 °C during 1-Hz sinusoidal 50% stretch.

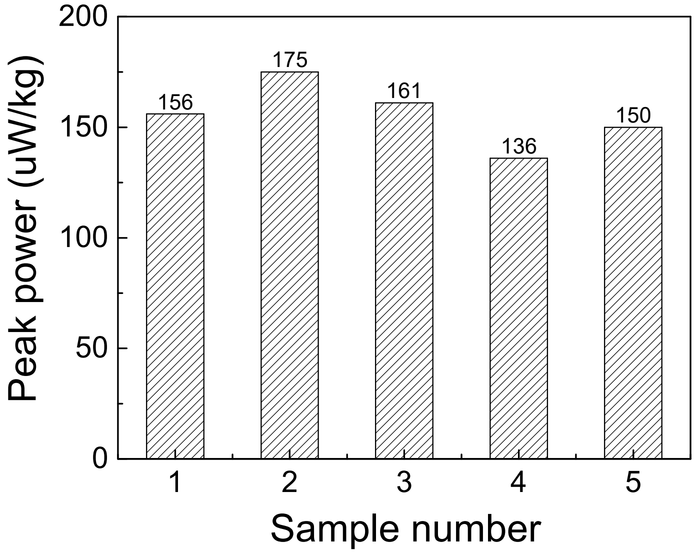

Figure S9. The maximum peak power of different 5 SECH sample.

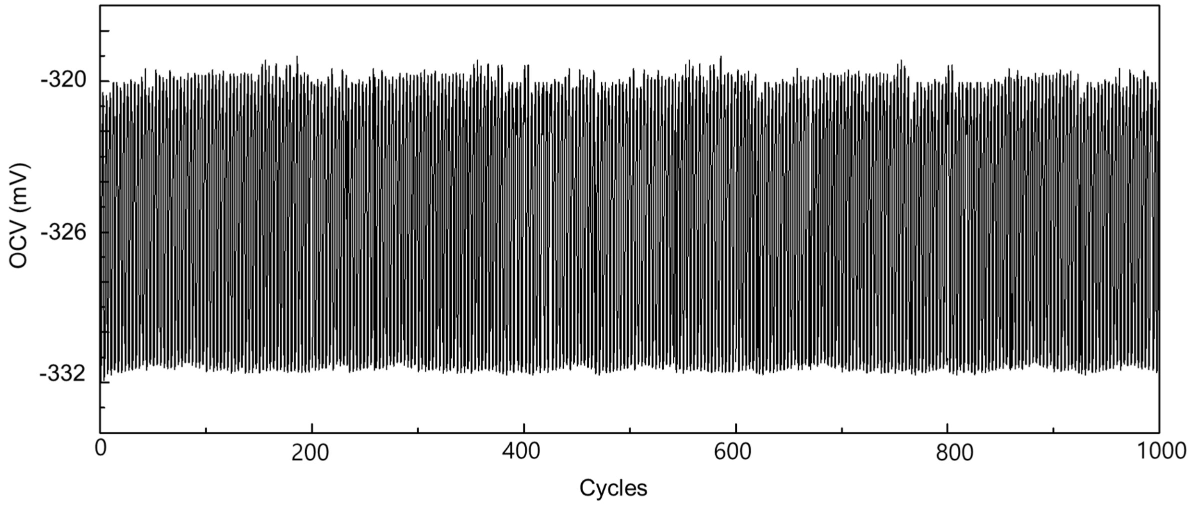

Figure S10. The open-circuit voltage during 1000 cycles.

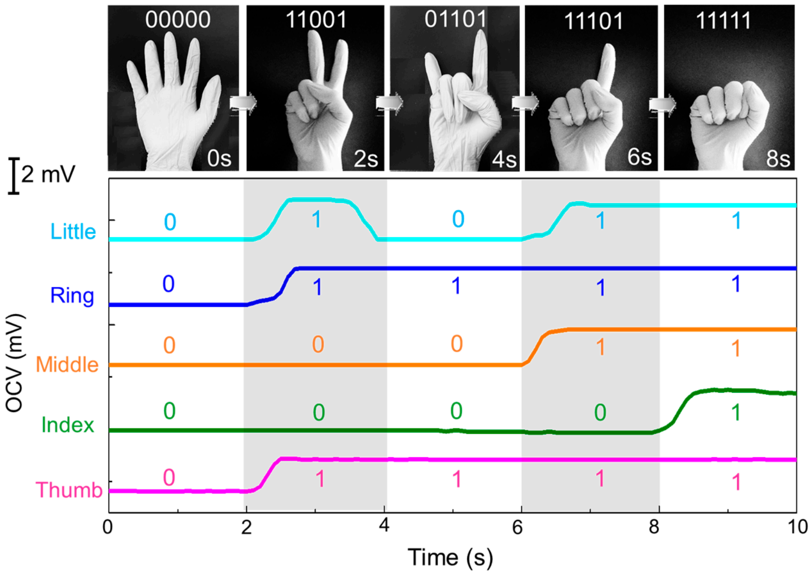

Figure S11. The open-circuit voltage changes versus time with hand configuration for hand signal. (inset) the photograph of hand configuration with binary number.
